# Research on High Precision and Deterministic Figuring for Shaft Parts Based on Abrasive Belt Polishing

**DOI:** 10.3390/ma12091389

**Published:** 2019-04-29

**Authors:** Xuelei Chen, Yifan Dai, Hao Hu, Guipeng Tie, Chaoliang Guan

**Affiliations:** College of Artificial Intelligence and Automation, National University of Defense Technology, 109 Deya Road, Changsha 410073, Hunan, China; 15954898151@163.com (X.C.); dyf@nudt.edu.cn (Y.D.); tiny_hh@139.com (H.H.); chlguan@nudt.edu.cn (C.G.)

**Keywords:** shaft parts, abrasive belt, deterministic, figuring, roundness error, polishing

## Abstract

A deterministic figuring method for cylindrical surface based on abrasive belt polishing is proposed in this study in order to improve the geometric accuracy of metal shaft parts. The principal motion of material removal is performed through the axial oscillation of the abrasive belt, and the different material removal at different positions can be obtained through servo control of the machine tool spindle by removing high error spots on the cylindrical surface and finally deterministically corrects the roundness error. An abrasive belt-based deterministic figuring device was built, and the figuring experiments were performed on the surface of steel workpieces 100 mm in diameter and 130 mm in effective length. The roundness errors of the entire workpiece after twice figuring iterations decreased nearly from the initial 3 μm to 1 μm, which preliminary verified the feasibility of this method. This deterministic figuring method is expected to break the machining accuracy limit and improve the rotation precision of the precision shaft parts such as the aerostatic spindle.

## 1. Introduction

Metal shaft parts have been widely used in various types of rotating machinery, ranging from heavy-duty, high-impact applications, such as the crankshaft of an internal combustion engine and turbine rotor, to high-precision, light load applications, such as precision spindles in cylindrical polishing machines [1]. The dimensional and geometric characteristics of shaft parts have a significant impact on the in-service performance of the component in any precision work. With the advancement of ultra-precision manufacturing technology such as the aerospace, aviation, and national defense industries etc., the geometric accuracy of some ultra-precision shaft parts should satisfy the order of 0.1 μm or even 0.01 μm [2]. The roundness of the shaft parts is a major factor determining the rotation accuracy. Its accuracy directly influences the matching and rotating precision of the shaft parts [3].

The existing ultra-precision cylindrical grinding is usually used to process high-precision shaft parts, whereas due to the restricted motion accuracy of the cylindrical grinder, the workpiece’s rotary precision has not been further improved for many years. Roundness error of sub-micron accuracy is usually acquired by manual polishing [4], which leads to high production cost and huge difficulty in improving accuracy. Thus, the demand for mass production cannot be met, and new shaft parts machining methods are urgently needed.

In recent years, there has been a tendency checking new tools and strategies for finishing metal parts, with repeatability and time/money savings being the main purposes. Adrián Rodríguez et al. studied brushing techniques using abrasive flexible tools to improve the surface finish and presented the brushing process to eliminate the spiral roughness pattern on metal brake discs and to optimize the surface of the machined part [5,6]. Abrasive belt polishing is also a flexible processing method for metal finishing. It has been proven by Rech and Moisan [7] that abrasive belt polishing removes the defects of the surface roughness induced by hard turning and leads to a very homogeneous surface. Accordingly, the abrasive belt polishing method has been widely used to enhance the surface quality. However, the deterministic correction of the shape accuracy of shaft parts based on abrasive belt polishing has been rarely studied.

In this study, a deterministic figuring method for the cylindrical surface based on the principle of optical polishing is proposed. The principal motion of material removal is performed through the axial oscillation of the abrasive belt. The different material removal at different positions can be achieved through servo control of the machine tool spindle, and finally, deterministically correct the roundness error. This study analyzed the assessment method of roundness error suitable for deterministic figuring, the depth model for material removal and the calculating method of the dwell time. Besides, property and figuring ability of removal function used for deterministic figuring is also theoretically analyzed. An abrasive belt-based deterministic polishing device was built, and figuring experiments were performed on the surface of steel workpieces 100 mm in diameter and 130 mm in effective length (total length of 200 mm).

## 2. Key Technology of Deterministic Figuring Based on Abrasive Belt Polishing

### 2.1. Principle of Deterministic Correction of Roundness Error

The deterministic figuring for the cylindrical surface is based on the CCOS (Computer Control Optical Surfacing) principle [8], i.e., under the determined removal function and the initial surface error, the dwell time of removal function at various points on the surface of the workpieces can be calculated by accurate dwell time algorithm. Subsequently, the numerical control system is utilized to control the polishing device to accurately implement the dwell time to achieve deterministic figuring of surface error.

**Figure 1:**
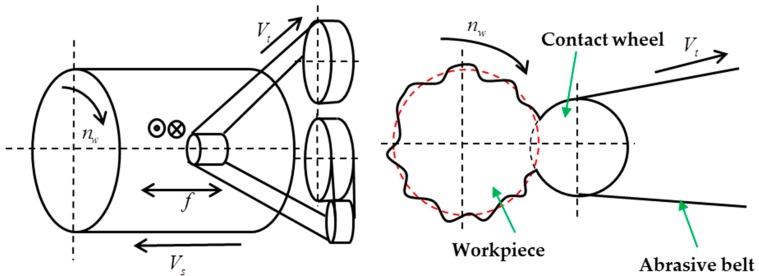
Schematic diagram for abrasive belt polishing.

The principle of deterministic figuring for the cylindrical surface is given in Figure 1. The contact wheel contacts with the workpiece under constant pressure and the abrasive belt can axially oscillate at a certain frequency *f* as driven by the contact wheel. The contact wheel can run through the workpiece axially at feed speed *V_s_*, and the workpiece’s rotary speed *n_w_* is precisely controlled by the machine tool servo spindle to achieve different dwell times at different positions. Accordingly, different material removal on the cylindrical surface can be obtained, thereby deterministically correct roundness error. To reduce or avoid the effect of belt abrasion during the figuring process, the abrasive belt feeds at speed *V_t_* to regenerate the abrasive grains in the contact area.

### 2.2. Depth Model for Material Removal

The material removal rate based on abrasive belt polishing can be described by a linear relation of the pressure and the velocity between the contact wheel and workpiece according to the Preston Equation [9]:

(1) $$R=\frac{dh}{dt}=k_{p}p(x,y)v(x,y)$$

where *R* is the material removal rate, *p*(*x,y*) denotes the pressure between the contact wheel and workpiece, *v*(*x,y*) is the relative velocity between the contact wheel and workpiece in contact area, *dt* is the polishing time, *dh* denotes the material removal depth of a certain point in the contact area, *k_p_* is the Preston coefficient related to machining conditions such as material properties, abrasives etc. other than the pressure and velocity.

**Figure 2:**
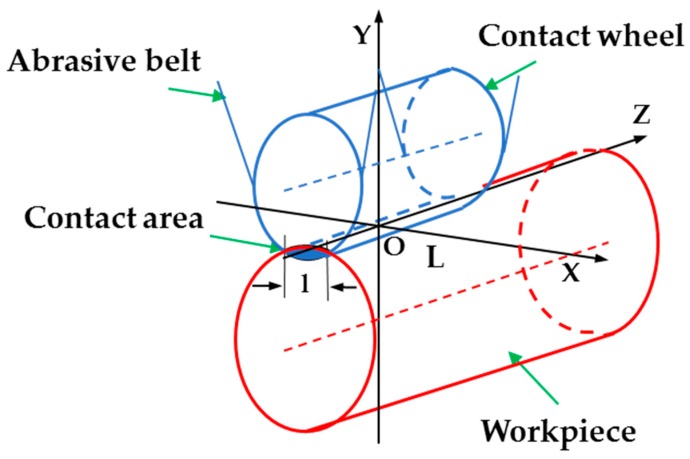
Contact between two cylindrical surfaces with parallel axes.

During the belt polishing process, the contact wheel is deformed against the cylindrical workpiece. The parameter L is the length of the contact area between the workpiece and the belt. The width of this area l depends on the applied force F and on the Young’s modulus of the contacting roller which is linked to the hardness of the material [10]. The workpiece used in the experiments is made of 45 steel, the Young’s modulus is 209 Gpa, and the contact wheel is made of rubber, its Young’s modulus is 0.0078 Gpa. In accordance with the Hertzian contact theory [11,12], the pressure in the plane of XOY in the contact area as showed in Figure 2 is distributed as an elliptical curve, and the pressure distribution can be expressed as:

(2) $$p(x)=-p_{0}\sqrt{1-{\left(\frac{x}{a}\right)}^{2}}$$

where *p*_0_ denotes the maximum pressure in the contact area, *a* is half the width of the ellipse contact area, i.e., l = 2 × *a.*

When the abrasive belt polishes the workpiece under constant pressure and oscillation frequency, the relative speed of a certain point in the contact area can be expressed as [13]:

(3) $$V_{v}=\pm 2\pi fAsin2\pi ft$$

where *A* denotes the oscillation amplitude of the belt, *f* is the oscillation frequency of the abrasive belt.

When the polishing time is *t*, the amount of material removed from a certain point in the contact area will be the cumulative removal amount on the abrasive belt at that point. According to the Preston equation:

(4) $$h(x)=\int k_{p}p(x)\left|V_{v}\right|dt$$

combining Equations (2), (3), and (4), the material removal in the contact area of the plane XOY can be expressed as:

(5) $$h(x)=4Atfk_{p}p(x)=-4Atfk_{p}p_{0}\sqrt{1-{\left(\frac{x}{a}\right)}^{2}}$$

The equation suggests that the material removal depth of the workpiece in the contact area of the plane XOY is linear with the oscillation amplitude, oscillation frequency, polishing time and pressure between the contact wheel and the workpiece.

### 2.3. Modeling of the Roundness Error Surface

**Figure 3:**
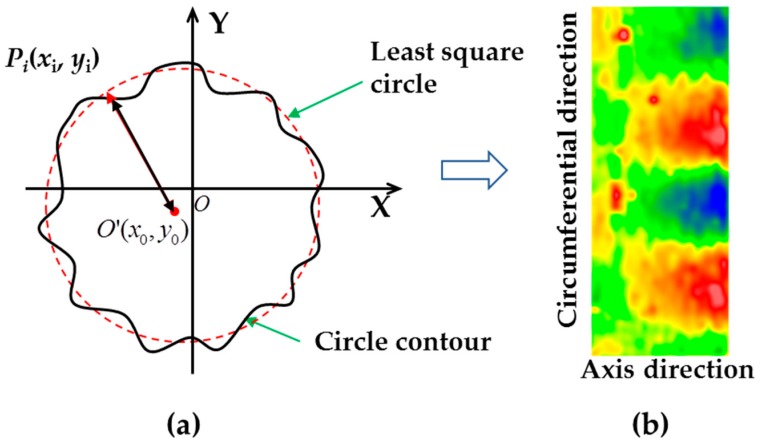
Modeling of roundness error surface: (**a**) Least square circle; (**b**) error points are interpolated to a roundness error surface.

The roundness information about a shaft part should be expressed on an error surface in order to achieve the deterministic figuring for a cylindrical surface. As shown in Figure 3a, assuming that the measured circle contour is on plane XOY, where *O* denotes the origin of the coordinates, $\left(R_{i}cos\theta_{i},R_{i}sin\theta_{i})(i=0,1,2\ldots m-1\right)$ are values in the cartesian coordinate system of the m measured points on plane XOY. The least square center *O′*(*x*_0_*, y*_0_) and the least square radius of circle *R* can be calculated by the following formula [14].

(6) $$\left\{ \begin{array}{l} x_{0}=\frac{2}{m}\sum_{i=1}^{n}R_{i}cos\theta_{i}\\ y_{0}=\frac{2}{m}\sum_{i=1}^{n}R_{i}sin\theta_{i}\\ R=\frac{1}{m}\sum_{i=1}^{n}R_{i} \end{array}\right.$$

The difference between the distance from each measured point *P_i_*(*x_i_, y_i_*) to the least square center of the circular contour *O′*(*x*_0_*, y*_0_) and the radius of the least square circle *R* can be expressed as:

(7) $$e_{i}=\sqrt{{\left(x_{i}-x_{0}\right)}^{2}+{\left(y_{i}-y_{0}\right)}^{2}}-R$$

*e_i_* is taken as the error value of each point. A plurality of circular contours along the axis direction are measured and the error values are interpolated along the circumferential direction and the axial direction, then the roundness error surface for deterministic figuring can be obtained. The horizontal direction represents circular contours in different axis positions, and the vertical direction represents different circumferential positions of a circular contour as shown in Figure 3b. Theoretically, when the error values converge to zero after figuring, an ideal absolute error plane can be obtained, and the circular contours of the cylindrical workpiece at different axial positions corresponding to the error surface are all absolute ideal circles.

### 2.4. Calculation of the Dwell Time

**Figure 4:**
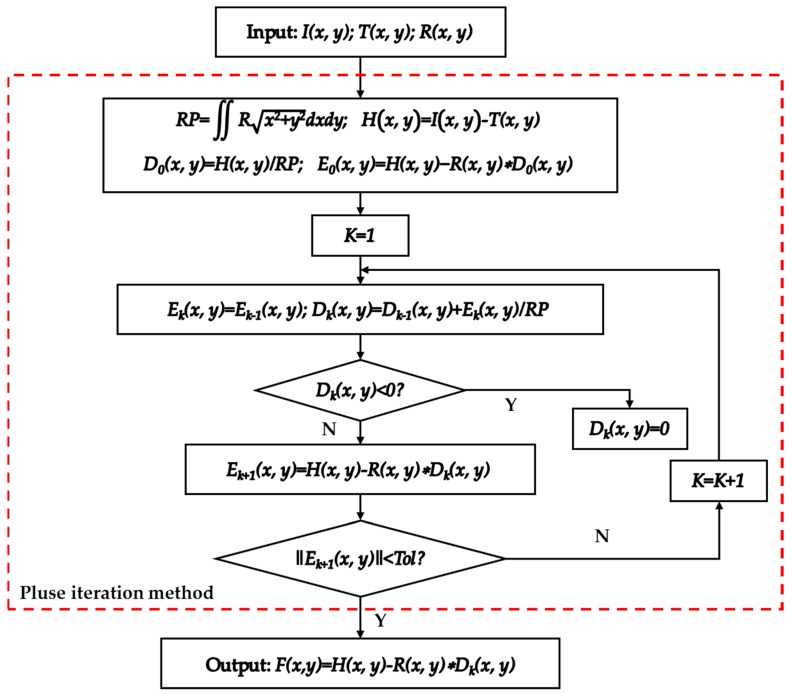
Flowchart for the pulse iteration method.

The material removed from the cylindrical surface can be obtained from the convolution of the dwell time and the tool removal function along the contour path [15]. By controlling the dwell time of the action area on the polishing track, the prescribed amount of material can be removed at each point. Figure 4 explains the pulse iteration method [16], where *I*(*x,y*) denotes the initial surface error, *T*(*x,y*) is the expected surface error after figuring, *H*(*x,y*) is the material removal distribution function, *R*(*x,y*) is the removal function, *Ω* is the action area of the removal function, *RP* is the removal pulse within the action area, *E_k_*(*x,y*) is the residual error, *D_k_*(*x,y*) is the dwell time calculated by pulse iteration method, *F*(*x,y*) is the surface error after simulation processing, and *Tol* is the convergence accuracy. The dwell time of the polishing area at different positions on the cylindrical surface can be calculated by pulse iteration method, and then the CNC (Computer Numerical Control) program that controls the implementation of the dwell time can be produced by the post-processing algorithm [17].

## 3. Experimental Set-Up

### 3.1. Figuring Experimental Device

**Figure 5:**
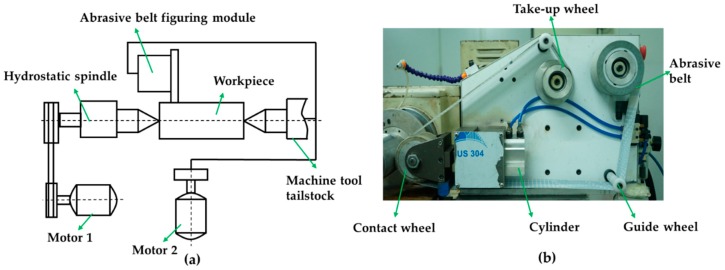
Figuring experimental device: (**a**) Schematic diagram of abrasive belt-based figuring system; (**b**) composition of the abrasive belt-based figuring module.

Abrasive belt polishing consists of pressing an oscillating abrasive polyester backed film against a rotating workpiece surface with a defined pressure by means of a contact wheel. The schematic diagram of abrasive belt-based figuring device is given in Figure 5a. The abrasive belt polishing module and the CNC module are installed on the machine tool of the hydrostatic spindle. As shown in Figure 5b, the abrasive belt polishing module consists of a contact wheel, an abrasive belt, a cylinder, a take-up wheel, a guide wheel, etc. The cylinder can eject and retract the contact wheel and further ensure the pressure between the contact wheel and workpiece remains constant during the polishing process. An elastic polishing area is formed between the abrasive belt and the workpiece under the extrusion between the rubber contact wheel and the workpiece. The belt can be driven by a motor to achieve high-frequency oscillation in the axial direction. The axial oscillation of the belt + contact wheel system is a key parameter of this polishing process in order to achieve material removal. Besides, fresh grains are introduced continuously and worn grains are eliminated at the same time during the polishing process [18].

The workpieces mounted on the machine tool can achieve circumferential rotation, and the abrasive belt polishing module mounted on the slide carriage is capable of achieving axial feeding, which allows the polishing area to traverse the entire cylindrical surface for machining. *Z* and *C* axes of the machine tool can be linked, thereby controlling the rotational speed of *C* axis and the feed rate of *Z* axis allows for implementing different dwell times at different positions on cylindrical surface to achieve the deterministic figuring of the cylindrical surface.

### 3.2. Roundness Error Measuring Device

**Figure 6:**
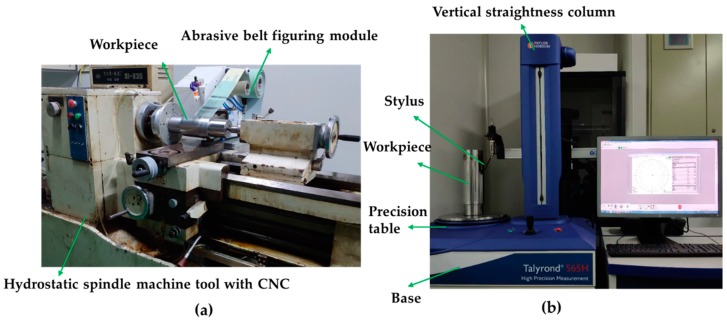
Machining and measuring device: (**a**) The abrasive belt-based figuring device; (**b**) the cylindricity measuring instrument.

The Taylor 565H type cylindricity measuring instrument, as shown in Figure 6b, is employed to measure the roundness of the shaft part. The axial and radial rotation motion accuracy of the instrument are both around 30 nm, and the straightness of its column is less than 70 nm. During the measurement, the stylus detects several equidistant circular contours from the bottom to top, and each circular contour is uniformly sampled to conclude the radial variation of the contour in the polar coordinate system [19]. Since these coordinate variation values include the shape error of the part, the systematic error of the cylindricity measuring instrument, the error caused by eccentricity and tilt of the part installation, etc. [20], the coordinate variation values are processed to obtain the roundness error surface by using the surface error modeling method introduced above.

## 4. Removal Functions for Deterministic Figuring of the Cylindrical Surface

### 4.1. Parameters Optimization of the Removal Function

The workpiece is fixed on the machine tool without any rotation, and the abrasive belt polishes the workpiece under a constant oscillation frequency and pressure for a certain time. Besides, the abrasive grains are ensured to be stable and timely updated during the polishing process within the contact area. The surface contours of the workpiece are measured before and after the polishing, and then the material removal distribution during polishing time can be obtained by subtraction of the two surface contours.

As mentioned before, the material removal rate of abrasive belt polishing is influenced by many factors. To study the effects of the grain size of the abrasive belt, the belt oscillation frequency and the pressure between the workpiece and the contact wheel on the material removal rate of abrasive belt polishing, three-factor three-level orthogonal experiments were performed to optimize the three independent critical parameters.

**Figure 7:**
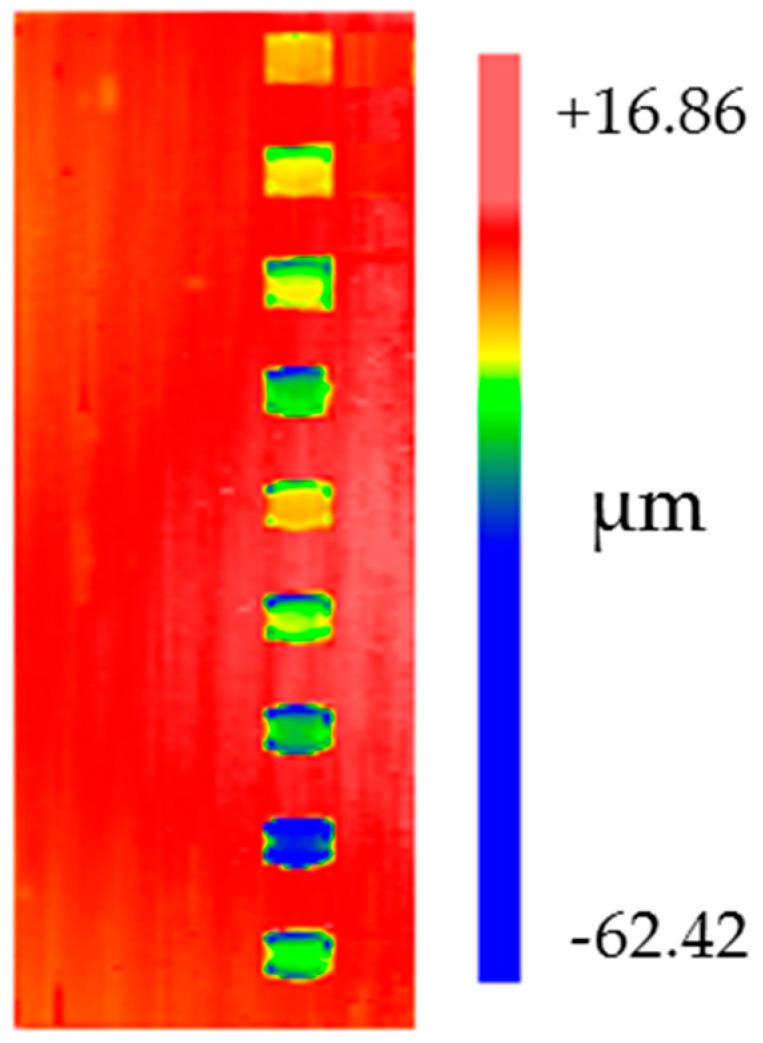
Removal functions under different parameters.

On the surface of the cylindrical workpiece made of 45 steel and had been prepared by precision turning, removal functions were made per 40 degrees in the circumferential direction using the factor level listed in Table 1 as experimental parameters. The polishing time for each group of parameters was 15 min. The surface contour of the workpiece is measured using the cylindricity measuring instrument before and after the experiment, and then the removal functions under different parameters are given in Figure 7.

**Figure 8:**
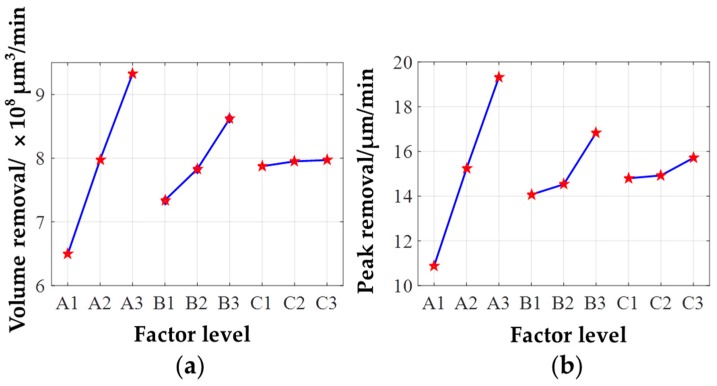
Removal rate under different factor levels: (**a**) Volume removal rate; (**b**) peak removal rate.

The curves of the average peak and volume removal rate of different removal functions under different factor levels are plotted in Figure 8. According to the result, the effect of the three factors on the removal rate is approximately linear, which verifies the above theoretical analysis of the removal function. The effect of different parameters on peak removal rate and volume removal rate is almost the same, suggesting that the shape of the removal function can be kept stable while ensuring stable removal efficiency.

**Figure 9:**
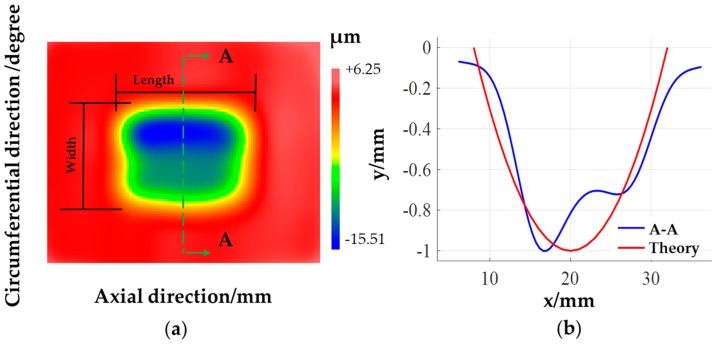
Removal function under optimization parameters: (**a**) Shape of removal function; (**b**) normalized contour of the removal functions.

According to the range R analysis, the grain size of the abrasive belt affects the material removal rate most significantly. The greater the grain size and the higher belt oscillation, the higher the material removal rate will be. However, the larger the grain size of the abrasive belt, the worse the surface quality of the workpiece will be after experiment [21]. In the meantime, since the machine tool used for machining performs the flexible synchronous belt transmission, if the selected pressure for deterministic figuring is too large, whether the loss of machine tool tracking accuracy would occur during the deterministic figuring process cannot be guaranteed. Thus, combining the above analysis, from the perspective of small error accumulation, good surface quality, and high machining efficiency, the optimal combination of parameters is given in Table 2. Besides, the removal function produced under these parameters is shown in Figure 9a.

The volume material removal rate of this removal function is 5.82 × 10^8^ μm^3^/min, the peak material removal rate is nearly 1.33 μm/min, the contour length in the axial direction is 30 mm, which is almost equal to the sum of the contact wheel width and the oscillation amplitude. The contour width in the circumferential direction is about 25 mm, and *a* in the theoretical analysis above is taken as 12 mm. The normalized contour and theoretical contour at the axial symmetry position of the removal function are plotted in Figure 9b. According to the Hertz contact theory, the material removal depth curve is theoretically parabolic, but due to the installation error, the workpiece axis and the machine tool tips connection are not in the same horizontal plane, so that the pressure distribution and the material removal depth is not uniform. The requirements for workpiece alignment are not high using this figuring method. Deterministic figuring process can be achieved as long as the contact state between the contact wheel and the workpiece during the actual figuring process is consistent with the removal function production process. The results show that the contour shape of the removal function obtained by the experiment is similar to the normalized contour shape of the theoretical analysis.

### 4.2. Stability of Material Removal Rate

**Figure 10:**
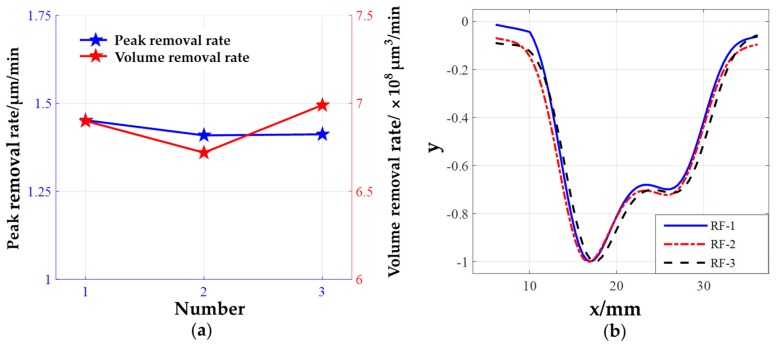
Removal function stability: (**a**) The variation curve of the peak and volume removal rate; (**b**) the normalized contour of different removal functions.

Removal function stability refers to the removal efficiency of removal function and the stability of geometric shape within a certain figuring time [22]. High material removal rate stability in a long-time polishing process is necessary for deterministic figuring. To verify the stability and repeatability of the removal function, experiments on 45 steel cylindrical workpieces were performed three times under the optimal experimental parameters as listed in Table 2, and the polishing time for each experiment was 15 min. The 45 steel cylindrical workpiece was initially prepared by precision turning. The variation curves of the peak and volume removal rate of different removal functions are plotted in Figure 10a, the stability of the volume removal rate is about 3.02%, and the stability of the peak removal rate is about 3.93%. The removal function profiles at their axially symmetric positions are extracted to compare their contour shape. Figure 10b shows the normalized profiles of different removal functions under the same parameters, and RF-X represent different removal functions in the plot. The ratio of the maximum deviation of the contour width of the three removal functions to the maximum contour width is calculated to characterize the shape change rate. The absolute value of the ratio is less than 5%. According to above results, the removal function of abrasive belt-based figuring exhibited excellent stability and repeatability, which verifies the removal function for deterministic machining of shaft parts can meet the figuring requirements for high efficiency and high material removal stability.

### 4.3. Figuring Ability of the Removal Function

**Figure 11:**
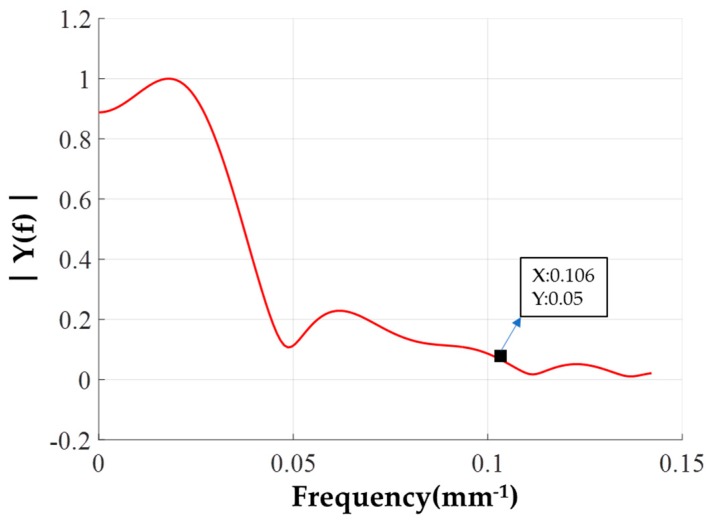
The normalized magnitude spectrum of the removal function contour.

Figuring ability of a removal function refers to the range of spatial frequency error that can be corrected [23]. Xuhui Xie [24] and Xusheng Zhou [25] et al. highlighted that the cutoff frequency of the removal function is the corresponding frequency at which the normalized amplitude spectrum of the removal function contour drops to 5% of the peak value. The removal function is capable of effectively correcting the surface error components below this cutoff spatial frequency. Figure 11 shows the normalized magnitude spectrum of the removal function contour, and the corresponding frequency is about 0.11 1/mm when the amplitude spectrum dropped to 5% of the peak. Thus, the removal function made under the optimal parameters is considered to be able to effectively correct the surface error of spatial wavelength greater than 10 mm.

**Figure 12:**
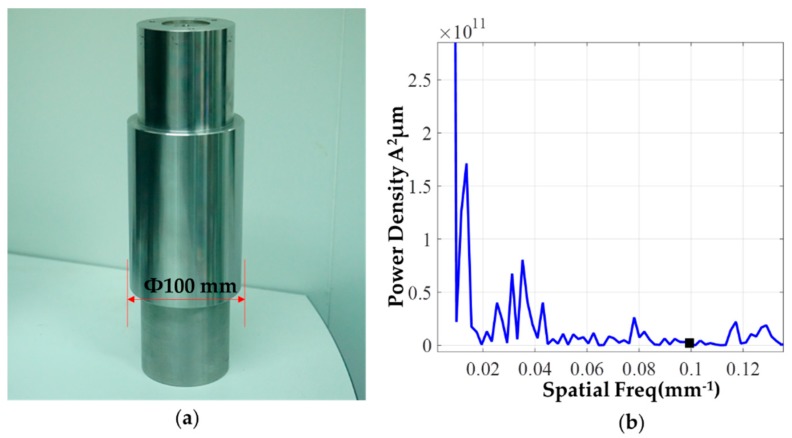
Workpiece and its profile spatial frequency: (**a**) Workpiece after the precision turning progress; (**b**) spatial frequency of a typical circular contour.

The actual contour of any machined surface always covers the geometrical error of the surface roughness profile, the waviness profile and the macroscopic shape profile [26]. Mathematical filtering can be applied to the roundness profile, to separate out the roughness from the general form. During the deterministic correction of roundness error, the shape error of low spatial frequency is emphasized. Through surface filtering, the mid-high frequency error components that cannot be corrected by the specific removal function can be filtered out, thereby reducing the actual machining residuals [27]. Figure 12a shows the workpiece after precision turning process and Figure 12b shows the spatial frequency distribution of the workpiece’s circular contour. It is shown that the circular contour covers a lot of low-frequency components, and the spatial wavelength of more than 90% of the contour is greater than 10 mm. Before using the surface error to calculate the dwell time, 10 mm Fourier low-pass filtering pre-processing is performed on the surface error in order to avoid excessive speed fluctuations during the figuring process. Based on the analysis of the figuring ability of the removal function, the removal function made under the optimal parameters can effectively correct the surface shape error after filtering.

## 5. Deterministic Figuring Experiments for Cylindrical Surface

To verify the feasibility of the deterministic figuring of the cylindrical surface based on abrasive belt polishing, experiments were performed on cylindrical workpieces using the abrasive belt figuring device built by our research group, as shown in Figure 6a. The workpiece is made of 45 steel, with a radius of 50 mm and the effective figuring length of nearly 130 mm (total length of 200 mm) and it was initially machined utilizing precision lathe.

The cylindricity measuring instrument as shown in Figure 6b was used to measure the roundness errors before and after experiments. The stylus detects a circular contour every 3 mm from bottom to top on the effective figuring length and total 44 circular contours were sampled. Then, the roundness error surface before and after figuring experiments can be obtained. Two iterations of figuring experiments were carried out utilizing the material removal distribution of the removal function made under the optimal parameters, and 10 mm Fourier low-pass filtering pre-processing was performed on the roundness error surface before using the surface error to calculate the dwell time. The first figuring time was about 130 min, and the second was about 50 min.

**Figure 13:**
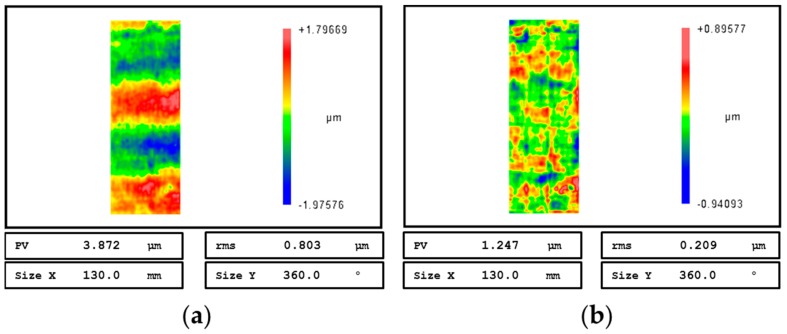
Roundness error surfaces before and after figuring: (**a**) The initial roundness surface error; (**b**) the roundness surface error after figuring process.

The initial and final roundness error surfaces are shown in Figure 13a and Figure 13b respectively. The results reveal that the PV value of the entire cylindrical surface after deterministic figuring for two iterations is improved from 3.872 μm to 1.247 μm, and the error convergence ratio is 3.11.

**Figure 14:**
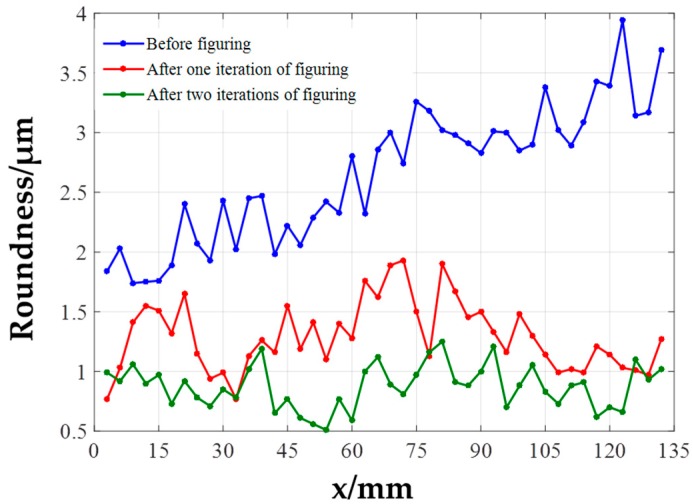
Roundness error at different axial positions.

The curves of roundness error value at different axial positions before and after figuring experiments are plotted in Figure 14. Roundness error curves before experiment i.e., after precision turning process, after figuring once and twice are shown in blue, red and green lines, respectively. According to the result, the roundness error at different axial positions of the experimental workpiece before figuring are overall larger than 1.5 μm, some even larger than 3 μm, and the roundness error gradually increases as the turning tool moves away from the clamping position result from clamping eccentricity error. After figuring for two iterations, the roundness error values are mostly less than 1μm, and the minimum roundness error value is 0.51 μm.

**Figure 15:**
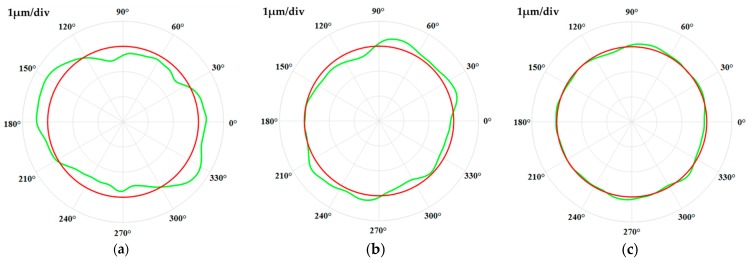
The shape of a circular contour at the same axial position: (**a**) Before figuring; (**b**) after figuring once; (**c**) after figuring two times.

The shape of the circular contours at the same axial position before and after figuring in the case where the scale is 1 micron per division are illustrated in Figure 15. Circular contour shape before figuring experiment i.e., after precision turning process is shown as Figure 15a, contour shape after figuring once and twice are shown as Figure 15b,c, respectively. Obviously, there exists a clamping eccentricity error, which makes the contour shapes elliptical after the precision turning process. The shape variations at the same axial position before and after the figuring experiment reveal that the high points on the circular contour gradually decreased, and the low points increased relatively, the elliptical contour is gradually close to the least square circle after the deterministic figuring for two iterations. This verifies that the deterministic correction of roundness errors can be obtained using abrasive belt-based figuring method.

According to above results, the deterministic figuring for the cylindrical surface based on abrasive belt polishing can achieve the deterministic correction of roundness error. However, similar to other deterministic machining methods, with the increase in the number of iterative machining, the deterministic figuring of abrasive belt will also introduce more mid-high frequency errors [28] and edge effect [29] to the cylindrical surface. In this study, the feasibility of deterministic figuring method for cylindrical surface to achieve the deterministic correction of roundness error is preliminarily verified on a simple abrasive belt polishing device built.

**Figure 16:**
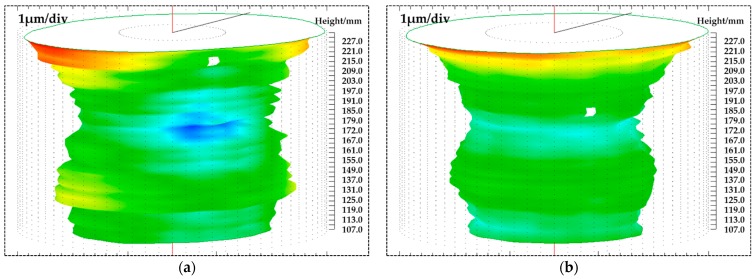
Cylindricity diagrams: (**a**) Before figuring process; (**b**) after figuring process.

The cylindricity diagrams before and after figuring are shown in Figure 16a and Figure 16b, respectively. The cylindricity errors before and after figuring operations are 9.17 μm and 7.79 μm. There is a slight improvement after figuring experiments. This paper mainly deals with the improving of roundness profile, there is no control over the cylindricity. While improving the roundness error, it is possible to worsen the cylindricity because there will be local changes in the axis of the cylindrical workpiece during roundness figuring process.

**Figure 17:**
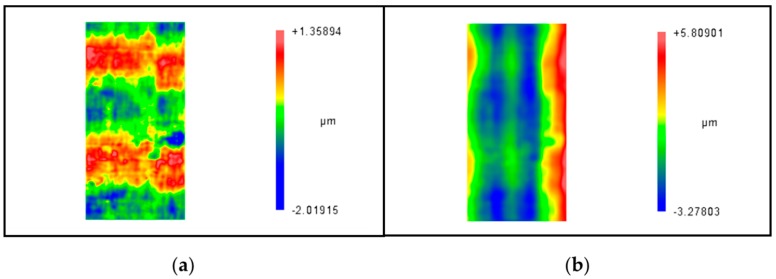
Error surfaces generated by the same workpiece. (**a**) Roundness error surface; (**b**) cylindricity error surface.

Generally speaking, the requirement for roundness is an order of magnitude higher than the cylindricity. In order to improve the whole geometry of the shaft parts, deterministic correction on the cylindricity error should be investigated. Roundness error and cylindricity error surfaces for deterministic figuring generated by the same cylindrical workpiece according to surface error modeling are quite different as shown in Figure 17a and Figure 17b respectively. The correct processing flow for the cylindrical workpiece deterministic figuring method is deterministic correcting the cylindricity to an appropriate accuracy first and then deterministic correct the roundness error. This paper only studies the latter, and the former needs further study.

## 6. Conclusions

In order to achieve the deterministic correction of roundness error, the study proposes a deterministic figuring method for cylindrical surface based on abrasive belt polishing and built a deterministic abrasive belt polishing device. The main conclusions are drawn as follows:

1. The axial oscillation of the abrasive belt can achieve material removal of the cylindrical surface, and the abrasive grains can be timely and stably updated in the polishing area during the deterministic figuring process. Through accurate modeling of surface error and accurate calculating of dwell time, the rotation speed of the *C* axis and feed rate of the *Z* axis can be obtained during the deterministic figuring process.

2. The volume material removal rate of the removal function made under the optimal parameters is 5.82 × 10^8^ μm^3^/min, and the peak material removal rate is nearly 1.33 μm/min. The relative change rate of volume material removal is about 3%, and the relative change rate of contour shape is less than 5%. The removal function exhibits excellent stability and repeatability and can be utilized to deterministic correction of the roundness error of shaft parts.

3. Verification experiments of deterministic figuring were performed on a cylindrical workpiece after the precision turning process. The PV value of the roundness error surface was improved from 3.872 μm to 1.247 μm after two iterations of figuring, with the convergence ratio of 3.11, which preliminary verified the feasibility of the deterministic machining principle for polishing metal shaft parts’ cylindrical surface.

4. After figuring twice, the mid-high frequency components on the workpiece surface increased, thereby affecting the convergence of roundness error. To achieve better shape precision and surface quality, machining technology that combines deterministic figuring and smoothing should be studied. In the meantime, the deterministic correction of the cylindricity error should be investigated, combining with the deterministic correction of the roundness error introduced in this paper, then the whole geometry of the shaft parts can be improved.

5. This deterministic machining with high spots removal method is expected to break through the precision limit of the machine tools. The experimental device needs further improving such as a thinner contact wheel should be designed and a force feedback module can be added in order to machine higher precision shaft parts. And this deterministic figuring method can also be extended to high-precision machining of metal workpieces such as crankshaft and cone, which needs further research.

## Figures and Tables

**Table 1:** Parameters of Orthogonal experiments.

Factor	A.Grain Size (μm)	B.Oscillation Frequency (Hz)	C.Pressure (MPa)	D.Time (min)
Level 1	9	5	0.25	15
Level 2	15	6	0.30	15
Level 3	30	7	0.35	15

**Table 2:** The optimal experimental parameters for removal function.

Grain Size (μm)	Polishing Frequency (Hz)	Pressure (MPa)
15	7	0.25
